# Highly mutable tandem DNA repeats generate a cell wall protein variant more frequent in disease-causing *Candida albicans* isolates than in commensal isolates

**DOI:** 10.1371/journal.pone.0180246

**Published:** 2017-06-29

**Authors:** Zhuo Zhou, Zoe Jordens, Shuguang Zhang, Ningxin Zhang, Jan Schmid

**Affiliations:** Institute of Fundamental Sciences, Massey University, Palmerston North, New Zealand; Louisiana State University, UNITED STATES

## Abstract

During adaptation to host environments, many microorganisms alter their cell surface. One mechanism for doing so is variation in the number of amino acid repeats in cell surface proteins encoded by hypermutable DNA tandem repeats. In the yeast *Candida albicans*, an opportunistic human pathogen, the gene *SSR1* encodes a GPI-anchored cell wall protein with a structural role. It contains two regions consisting of tandem repeats, almost exclusively encoding the amino acid pair Ser-Ala. As expected, the repeat regions make *SSR1* highly mutable. New *SSR1* alleles arose with a frequency of 1.11×10^−4^ per cell division in serially propagated cells. We also observed a large number (25) of *SSR1* alleles with different repeat lengths in a survey of 131 isolates from a global strain collection. *C*. *albicans* is diploid, and combinations of these allele generated 41 different *SSR1* genotypes. In both repeat regions, nonsynonymous mutations were largely restricted to one particular repeat unit. Two very similar allele combinations were largely restricted to one clade, clade 1. Each combination was present in ~30% of 49 infection-causing clade 1 strains, but one was rare (2%), the other absent in 46 infection-causing strains representing the remainder of the species (*P* < 0.00018 and 0.00004; Fisher’s exact test). These results indicate that both repeat regions are under selection and that amino acid repeat length polymorphisms generate Ssr1 protein variants most suitable for specific genetic backgrounds. One of these two allele combinations was 5.51 times more frequent, the other 1.75 times less frequent in 49 clade 1 strains that caused disease than in 36 commensal clade 1 strains (*P* = 0.0105; Chi^2^ test). This indicates that insertion and deletion of repeats not only generates clade-optimized *Ssr1p* variants, but may also assist in short-term adaptation when *C*. *albicans* makes the transition from commensal to pathogen.

## Introduction

Bacterial and eukaryotic pathogens use the high frequency of insertions and deletions in tandem repeat (TR) DNA in so–called contingency genes to rapidly and transiently adapt to specific host niches or to avoid the host’s immune response by altering expression or the amino acid sequences of the encoded proteins [1,2]. The genome of the yeast *Candida albicans*, an important opportunistic human pathogen [3], contains thousands of ORFs (TR-ORFs) in which DNA tandem repeats encode amino acid repeats [4,5]. Repeat number variation in TR-ORFs generates large numbers of alleles, and the resulting protein variants are functionally different, as inferred either from the nonrandom distribution of alleles or from direct functional analysis [6–17]. However, at least the vast majority of TR-ORFs do not seem to act as contingency genes. Specific alleles or allele combinations (*C*. *albicans* is diploid) are not typically associated with the niche from which a strain is isolated, but rather with specific clades, i.e. genetic backgrounds, [6–10,16,17]. Individual clades are generally not confined in distribution to specific geographical regions, body sites or types of candidiasis [18,19]. Thus these findings suggest that repeat number variation assists predominantly in long-term adaptation, optimizing proteins for a genetic background, plus possibly in increasing the overall rate of evolution of the proteome [5,16].

There is only very limited evidence suggesting possible associations of different TR-ORF alleles with different niches, such as commensalism versus disease [20]. Allele changes that are correlated with the transition from commensal to pathogen would be of great interest, not only because they would improve our understanding of this transition, but also because these could potentially assist in predicting the onset of candidiasis and thus early diagnosis of the disease, an important determinant of survival of life-threatening *C*. *albicans* bloodstream infections [3].

Many known contingency genes encode cell surface proteins [1,2]. To expand the search for *C*. *albicans* contingency genes we investigated the TR-ORF of a surface protein-encoding gene, *SSR1*, encoding a GPI-anchored cell wall protein, that plays a role in cell wall stability [21].

## Materials and methods

### Strains and culture methods

The set of strains used in this study represents a collection of 131 isolates, including 86 clade 1 isolates from 6 countries plus 46 isolates from the remainder of the species [18,22–25] (S1 Table; permission for use of these *C*. *albicans* isolates had been granted by the Massey University Human Ethics Committee; the table also lists an additional strain, the laboratory strain Sc5314, but this was not used in our analyses). All strains had only been cultured briefly before being stored at -80°C and were not extensively cultured as part of this work. The exception was one RIHO30 clone, which was serially transferred for 300 generations in YPD medium. The number of transfers required was calculated based on the increase in optical density at 600 nm measured with a spectrophotometer (NOVA TECH). This was followed by plating on YPD plates at ~100 CFU/plate, from which 60 single colonies were chosen to determine *SSR1* allele sizes by genotyping.

### Bioinformatic search for *SSR1* ORFs sequenced in other *Candida* strains and for conserved domains within Ssr1p

*SSR1* ORFs sequenced in other *C*. *albicans* strains and in *C*. *dubliniensis* were identified in a BLASTP search against the NCBI non-redundant protein sequence database, using the Ssr1p amino acid sequence lab strain SC5314 as a query. The same sequence was used to search for conserved domains in Pfam (http://pfam.xfam.org).

### Molecular biology methods

**Fig 1:**
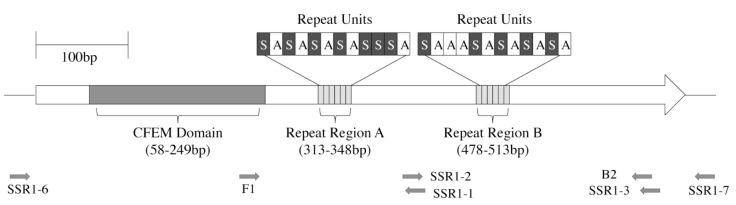
*SSR1* ORF as present in the SC5314 genome. Locations of the two repeat regions and a likely CFEM domain identified by us are indicated. Grey arrows indicate primers used (listed in Table 1).

*SSR1* repeat regions were PCR-amplified [26] using as a template a single colony either directly or 1 μl of a solution prepared by boiling the colony in 15μl of water for 4 minutes and removing debris and cells by spinning the mixture in the cold for 1 min at 13,000 x g [27]. Polymerase chain reactions contained, in a volume of 20μl (colony) of 25μl (boiled supernatant) [26] 1 unit of Taq DNA polymerase (Roche Diagnostics, Auckland, New Zealand), 200 μM of each dNTP (Roche), 10 pmol of each primer (Invitrogen/Gibco BRL) and 4 μl of Q-buffer (Qiagen Pty Ltd, Clifton Hill Vic, Australia). Primer sequences and PCR conditions are listed in Table 1 (positions of primers are also shown in Fig 1).

**Table 1:** Primers used and PCR conditions.

primer 1*	primer 2*	PCR conditions
**F1**	**B2**	3 min at 94°C
TGATGCTGATACTGCCATTT	AGCACCAAGACCAACCTTAG	30 x (40 sec at 94°C / 40 sec at 53°C / 50 sec at 72°C) 20 min at 72°C
**F1**	**SSR1-1**	2 min at 94°C
TGATGCTGATACTGCCATTT	AGAAGAAGCCTTGGCTGAAC	30 x (30 sec at 94°C / 30 sec at 55°C / 30 sec at 72°C) 20 min at 72°C
**F1**	**SSR1-3**	2 min at 94°C
TGATGCTGATACTGCCATTT	AGACCAACCTTAGCAGCAGAAC	30 x (45 sec at 94°C / 40 sec at 55°C / 30 sec at 72°C) 20 min at 72°C
**SSR1-2**	**SSR1-3**	2 min at 94°C
CTAGTTCAGCCAAGGCTTCTTC	AGACCAACCTTAGCAGCAGAAC	30 x (30 sec at 94°C / 30 sec at 55°C / 30 sec at 72°C) 20 min at 72°C
**SSR1-6**	**SSR1-7**	5 min at 96°C
GTCTATCGTTACTACTACTACC	AATAATTGCGAAGGGGGCAG	30 x (45 sec at 94°C / 40 sec at 55°C / 50 sec at 72°C) 10 min at 72°C
**M13F**	**M13 R**	3 min at 94°C
CCCAGTCACGACGTTGTAAAACG	AGCGGATAACAATTTCACACAGG	30 x (30 sec at 94°C / 30 sec at 63°C / 1 min at 72°C) 5 min at 72°C

* Location of *SSR1* primers is shown in Fig 1.

Repeat region lengths were determined by genotyping of amplicons, using primers listed in Table 1 (positions shown in Fig 1.) and product sizes were determined by the Genotyping for Microsatellite Analysis Service at the Alan Wilson Centre Genome Services (Massey University, Palmerston North, New Zealand), using a 3730 Genetic Analyzer, using the size standard 500 LIZc (Applied Biosystems) and software *Peak Scanner* (Applied Biosystems). For *SSR1*-heterozygous *C*. *albicans* strains, the repeat unit sizes were inferred by generating PCR products containing both repeat regions as well as individual regions (Table 1, Fig 1). Genotyping-derived fragment sizes were converted into 6bp repeat numbers based on sequencing of select *SSR1* ORFs (Peak Scanner’s estimates of the size of a 6 bp unit inferred from these comparisons were close to theoretical expectations, namely 6.03± 0.02 bp).

For determining the sequences of entire *SSR1* ORFs, these were amplified using primers SSR1-6 and SSR1-7 (Table 1) and ligated into the pLUG®-Multi TA cloning vector (iNtRON). The constructs were used to transform *E*. *coli* DH5α [26]. The inserts were amplified using vector sequences M13F and M13R (Table 1) on an ABI 3730 DNA Analyzer Alan Wilson Centre Genome Services (Massey University, Palmerston North, New Zealand). All nucleotide sequences have been submitted to GenBank (Accession numbers KY569347 to KY569357).

## Results and discussion

### The *SSR1* ORF contains two highly variable tandem repeat regions encoding amino acid repeats that are under selection

The *C*. *albicans* gene *SSR1* encodes a GPI-anchored cell wall protein with a CFEM domain, (a cysteine-containing domain common to fungal surface proteins; InterPro IPR008427), that plays a role in cell wall stability [21]. In the SC5314 reference genome (http://www.candidagenome.org/), the *SSR1* ORF contains two tandem repeat regions, separated by 200 bp of nonrepetitive DNA. Both repeat regions encode a series of amino acid pairs, mainly Ser/Ala (Fig 1). Both regions were also present in all of 131 strains from an international collection of infection-causing and commensal isolates that we tested (S1 Table), and in 18 *C*. *albicans SSR1* sequences in Genbank (S2 Table), but the length of the regions varied. By genotyping, sequencing and analyzing published Genbank sequences we identified 8 different lengths of repeat region A (range 6–13 repeats) and 13 different lengths of repeat region B (range 4–31 repeats), generating 32 alleles (S1 and S2 Tables). *C*. *albicans* is diploid [28] and the 25 different alleles we identified in the 131 isolates from our collection combined into 41 different allele combinations (S1 Table).

**Fig 2:**
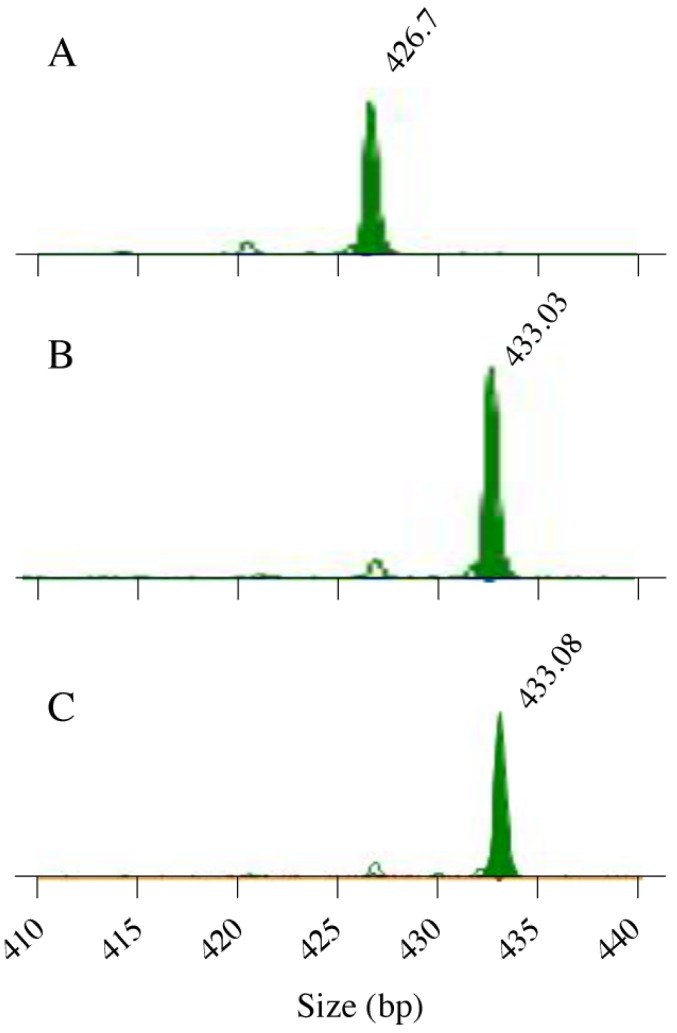
Genotyping of repeat region B showing mutant alleles after 300 generations. (A) Size of one of the original repeat region B amplicons in strain RIHO30 (31 repeats) as displayed by the software Peak Scanner. (B),(C) Repeat region B amplicons, increased in size by 6 bp (by 1 repeat unit, to 32 repeats), from 2 different colonies after serial propagation of the strain for 300 generations.

This diversity was not unexpected, given the high mutation rate of repetitive DNA [29], which we confirmed directly for the *SSR1* repeats by serially propagating one of the strains, RIHO30. RIHO30 is homozygous for an *SSR1* allele with 6 repeat units in region A and 31 units in region B. After 300 generations in two of sixty colonies tested, one allele had undergone a region B expansion (from 31 to 32 repeats; Fig 2) This is equivalent to a mutation rate, in strain RIHO30, of 1.11×10^−4^ mutations in *SSR1* per cell division, 5.5×10^−5^ mutations per allele, 2.7×10^−5^ mutations per repeat region and 1.5 x 10^−6^ mutations per base pair in repeat regions (6 bp addition to 6 x 37 x 2 base pairs in 1/30 cells after 300 generations). This rate exceeds the estimated frequency of point mutations of ~3 x 10^−10^ per bp per division [30,31] by several orders of magnitude.

**Fig 3:**
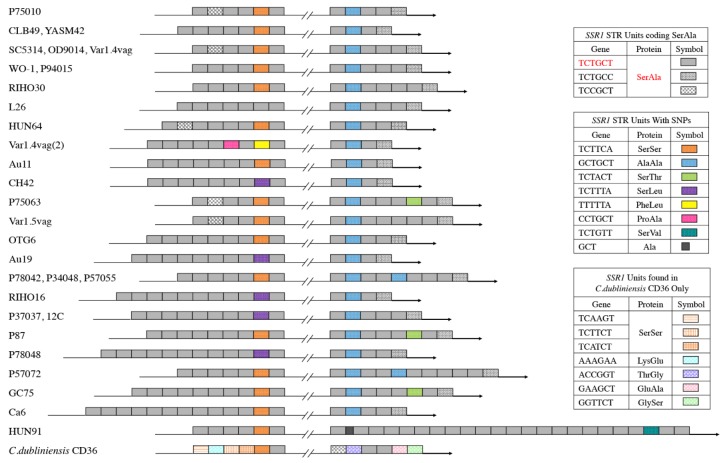
*SSR1* allele region structures. Predicted amino acid sequences of repeat regions of 23 different *SSR1* alleles and the repeat region from the *C*. *dubliniensis* CD36 ortholog. Different shadings of repeat units indicate different versions of DNA and amino acid repeats, with colored units representing nonsynonymous mutations, leading to amino acid pairs other than SER/ALA. Alleles of stains CLB49, YASM 42, OD9014, Var1.4vag (both alleles), RIHO30, HUN64, Au11, CH42, Var1.5vag, OTG6, Au19, RIHO16, and HUN91 were sequenced as part of this study, while the other alleles were from the *C*. *albicans* genome database (http://www.candidagenome.org/) or were identified by BLAST searches.

Aside from length polymorphisms in published alleles and in 14 alleles we sequenced, we observed point mutations that distinguished repeat units from each other. However nonsynonymous point mutations were distributed in a nonrandom fashion, being almost completely restricted to the second-to-last repeat unit in region A and the second repeat unit in region B (Fig 3). This indicates that the amino acid repeats are under selection, i.e. that these regions are phenotypically relevant, as does the retention of these regions after the divergence of *C*. *albicans* and *C*. *dublinensis* (Fig 3). They might contribute to the functionality of the Ssr1 protein or to cell-surface mediated interactions with the host environment.

### High prevalence of specific allele combinations in clade 1 strains

**Fig 4:**
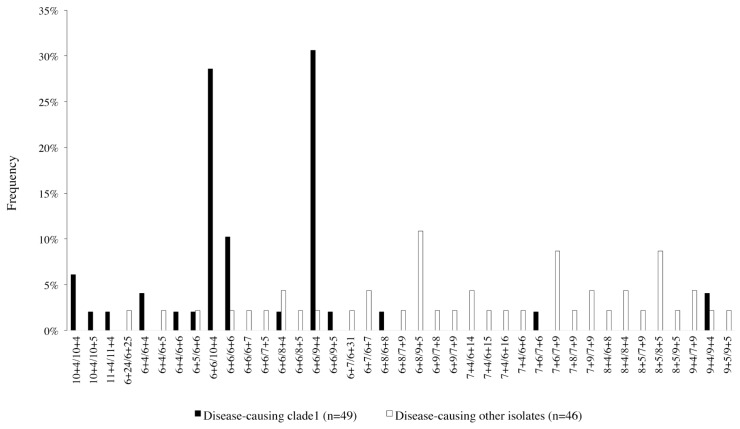
Frequency of *SSR1* genotypes in disease-causing isolates. Allele combinations are labeled with repeat numbers in allele 1 (Region A + Region B) followed by repeat numbers in allele 2 (Region A + Region B); alleles are sorted so that allele 1 is the smaller allele (when alleles are of the same size the allele with the larger region A precedes the allele with the smaller region A). Genotypes for 49 disease-causing clade 1 isolates and 46 other disease-causing isolates are shown.

We assessed if among isolates belonging to the genetically most homogeneous *C*. *albicans* clade, clade 1 [18,32], particular allele combinations were overrepresented, relative to the remainder of the species. We did this initially for the 95 disease-causing isolates (since the set of strains we had chosen to analyse included commensal isolates only from clade 1). We identified two very similar allele combinations that were very frequent in clade 1 and rare in other strains. One (6+6/9+4; one allele with 6 repeats in regions A and B, one with 9 in A and 4 in B) was present in 15 out of 49 disease-causing clade 1 isolates but only in 1/46 other isolates (*P* = 0.00018; Fisher’s exact test) and another (6+6/10+4) was present in 14 out of 49 clade 1 isolates and in 0/46 other isolates (*P* = 0.00004; Fisher’s exact test; Fig 4). The most likely explanation of this result is that clade1 genetic background selects for these alleles since their frequency was similar regardless of geographical region, the site of isolation, age or sex of patients for which clade 1 isolates had been obtained (S1 Table). Given the mutation rate of the repeat regions and the time of divergence of clades [33], it is unlikely that the uniform increased frequency of these allele combinations in clade 1 was merely generated by genetic drift.

### The frequency of allele combinations differs between disease-causing and commensal clade 1 isolates

**Fig 5:**
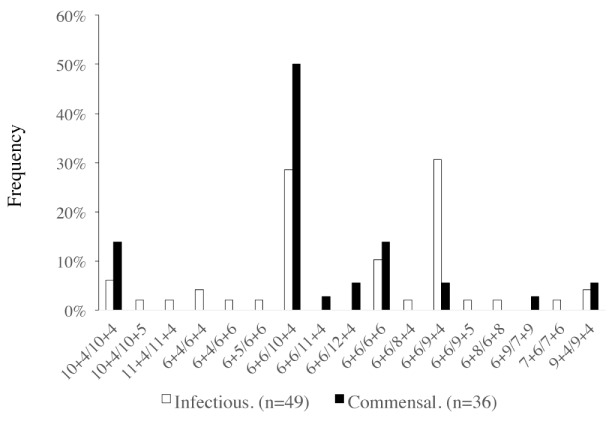
Frequency of genotypes in 36 commensal and 49 disease-causing clade 1 isolates. Allele combinations are labeled with repeat numbers in allele 1 (Region A + Region B) followed by repeat numbers in allele 2 (Region A + Region B) alleles are sorted so that allele 1 is the smaller allele (when alleles are of the same size the allele with the smaller region A precedes the allele with the larger region A).

The genetically fairly homogeneous clade 1 strains also provided an opportunity to explore if, within a given genetic background, different interactions with the host might select for different *SSR1* alleles. Indeed, while jointly the allele combinations 6+6/9+4 and 6+6/10+4 accounted for a similar percentage (60%) of *SSR1* genotypes in commensal isolates and disease-causing clade 1 isolates (55.6% and 59%, respectively), the allele combination 6+6/9+4 was 5.51 times more frequent in 49 clade 1 strains that caused disease than in 36 commensal clade 1 strains, and the combination 6+6/10+4 was1.75 times less frequent (*P* = 0.0105; Chi^2^ test; Fig 5). No other patient or isolate characteristics were associated with significant differences in in the frequencies of these two alleles (S1 Table). Also, while the size of subsamples was too small to establish statistical significance, the observed frequency of the 6+6/9+4 allele type was higher in pathogenic clade 1 isolates from both males and females (S1 Fig), and when only adults age 20–69 were considered (S2 Fig). It was comparable between clade 1 isolates from nonsterile and sterile sites of infection (S3 Fig).

In summary, our results support a role of the high frequency of mutation of the *SSR1* repeats in the generation of clade-specific variants of the Ssr1 protein. An asymmetric distribution of nonsynonymous mutations and an association of some Ssr1 variants with clade 1, in spite of the high mutation rate of *SSR1* TR repeats, indicates that the TR-encoded amino acid repeats are under selection and thus phenotypically relevant. Presumably the amino acid repeats optimize the protein for different genetic backgrounds, as is also that case for many other amino acid repeat-containing proteins [16]. Our data indicate that, in addition, commensal and pathogenic interaction with the human host may lead to selection of different *SSR1* alleles. Disease-causing strains do not represent separate genetic lineages [18,34] and candidiasis is often caused by endogenous commensal strains[35,36]. Thus our data suggest that alterations in proteins encoded by repeat-containing genes such as *SSR1* may be part of the transition of *C*. *albicans* from commensal to pathogen. If so, changes in *SSR1* would not necessarily play a primary role in pathogenesis, but could be a secondary adaptation to disease-related changes in the physiology of the host or *C*. *albicans* or mutational changes in other *C*. *albicans* TR-ORFs.

Unfortunately the functional significance of changes in *SSR1* in human disease cannot be demonstrated directly by transforming a laboratory strain with the 6+6/9+4 and other allele combinations and assessing if the former increases its virulence in an animal model, for a number of reasons. One is that the results of *C*. *albicans* virulence assessments in animal models conflict with outcomes in humans[37], and that different strain types (and thus alleles) are favoured by selection in animals compared to humans [38]; thus virulence differences between *SSR1* alleles in the animal model (or their absence) would be difficult to interpret in the context of human disease, even if changes in *SSR1* did play a primary role in human disease. Secondly, which TR-ORF alleles are selectively advantageous depends on the genetic background of the strain (this study,[16,39]); thus the 6+6/9+4 allele combination may not necessarily be compatible with the genetic background of a given laboratory strain, especially since such strains’ genomes differ from those of natural isolates[10,16,40]. Lastly, given the large number of TR-ORFs in the *C*. *albicans* genome [4,5], it seems likely that mutational changes in TR-ORFs during pathogenesis are not restricted to *SSR1*, and that changes in *SSR1* or indeed any given TR-ORF are only advantageous if they are matched with compatible changes in other TR-ORFs.

If the latter is correct, the significance of changes in TR-ORFs in pathogenesis may however be verifiable in the future by whole genome and amplicon sequencing of large numbers of TR-ORFs in commensal and disease-causing isolates, in particular of colonizing and blood stream isolates from the same patients. Such analyses would then reveal a limited number of sets of concerted changes in multiple TR-ORFS correlated with the transition from commensalism to pathogenesis. Since such correlations involving multiple TR-ORFs would be extremely unlikely to arise by chance they would represent strong, albeit indirect evidence that mutational change in TR-ORFs is part of *C*. *albicans*’ transition from commensal to pathogen.

## Supporting information

S1 Fig Frequency of *SSR1* genotypes in disease-causing and commensal clade 1 isolates in males and females. Numbers in brackets are the numbers of isolates in each category.
(TIFF)

S2 Fig Frequency of *SSR1* genotypes in disease-causing and commensal clade 1 isolates in patients of different age; teens, adults aged 20–69 and patients older than 69. Numbers in brackets are the numbers of isolates in each category.
(TIFF)

S3 Fig Frequency of *SSR1* genotypes in disease-causing clade 1 isolates from sterile and nonsterile sites. Numbers in brackets are the numbers of isolates in each category.
(TIFF)

S1 Table Isolates and their *SSR1* repeat region size combinations. (DOCX)

S2 Table *SSR1* repeat region sizes in published sequences from other strains. (DOCX)
