# Supplementary figures and images for: Highly mutable tandem DNA repeats generate a cell wall protein variant more frequent in disease-causing Candida albicans isolates than in commensal isolates
Source: PLoS One. 2017 Jun 29;12(6):e0180246. doi: 10.1371/journal.pone.0180246 (PMC5491155; doi:10.1371/journal.pone.0180246)

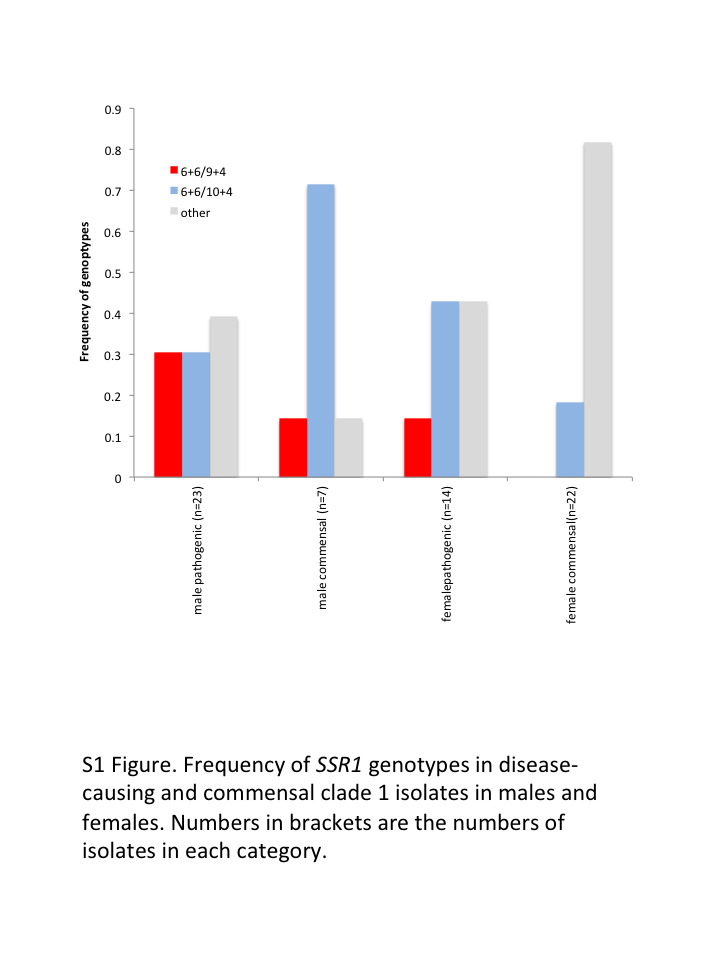

Supplement: S1 Fig — Numbers in brackets are the numbers of isolates in each category. (TIFF) [file pone.0180246.s001.tiff]

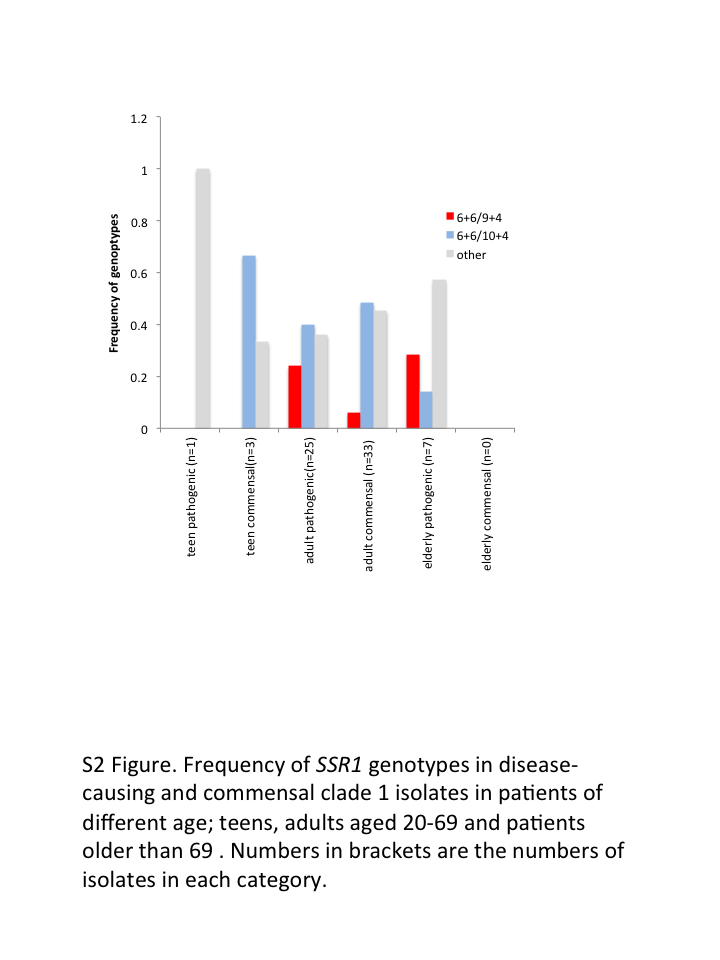

Supplement: S2 Fig — Numbers in brackets are the numbers of isolates in each category. (TIFF) [file pone.0180246.s002.tiff]

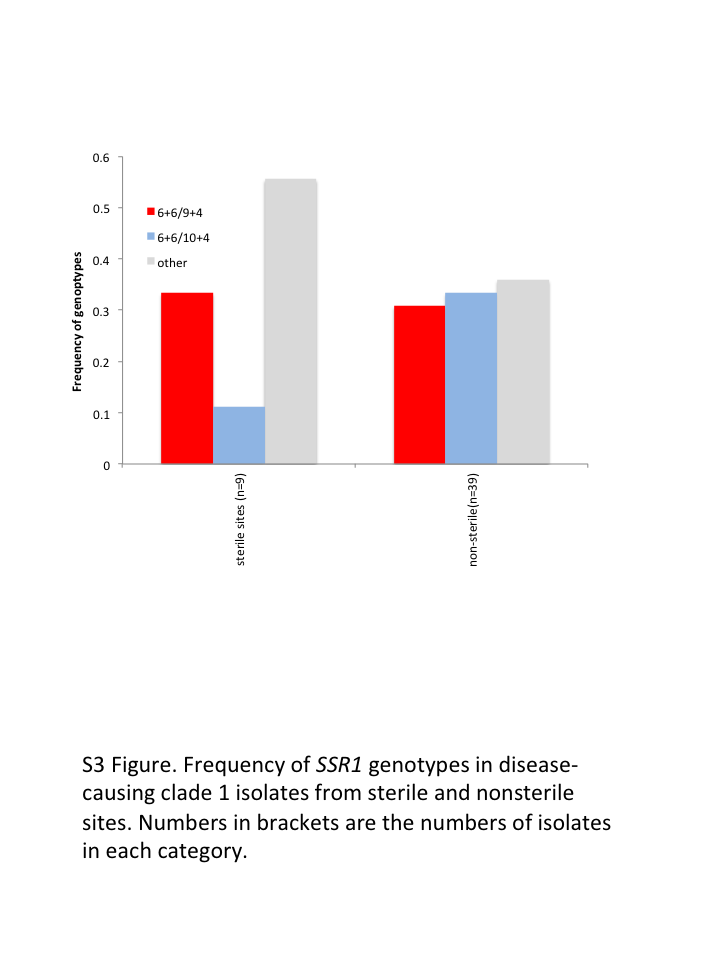

Supplement: S3 Fig — Numbers in brackets are the numbers of isolates in each category. (TIFF) [file pone.0180246.s003.tiff]
