# Supplementary material for: Highly mutable tandem DNA repeats generate a cell wall protein variant more frequent in disease-causing Candida albicans isolates than in commensal isolates
Source: PLoS One. 2017 Jun 29;12(6):e0180246. doi: 10.1371/journal.pone.0180246 (PMC5491155; doi:10.1371/journal.pone.0180246)
Supplement: S1 Table — (DOCX) [file pone.0180246.s004.docx]

**S1Table. Isolates and their *SSR1* repeat region size combinations**

| Strains^a^ | Group^a^ | infectious/ commensal | Site of isolation^b^ | Country of origin | Number of repeats ^c^ | | | | | | | Accession number(s) |
| --- | --- | --- | --- | --- | --- | --- | --- | --- | --- | --- | --- | --- |
|  |  |  |  |  | allele combination | allele1 region 1 | allele1 region 2 | allele2 region 1 | allele2 region 2 | Allele 1 | Allele 2 |  |
| hp31an | clade 1 | Commensal | a | USA | 10+4/10+4 | 10 | 4 | 10 | 4 | 10+4 | 10+4 |  |
| Jam-2c | clade 1 | Infectious | a | USA | 6+6/9+4 | 6 | 6 | 9 | 4 | 6+6 | 9+4 |  |
| W26 | clade 1 | Infectious | a | New Zealand | 6+6/6+6 | 6 | 6 | 6 | 6 | 6+6 | 6+6 |  |
| AU1 | clade 1 | Infectious | r/o | New Zealand | 6+6/6+6 | 6 | 6 | 6 | 6 | 6+6 | 6+6 |  |
| COD21 | clade 1 | Commensal | r/o | UK | 6+6/6+6 | 6 | 6 | 6 | 6 | 6+6 | 6+6 |  |
| cour-c | clade 1 | Infectious | r/o | USA | 6+6/10+4 | 6 | 6 | 10 | 4 | 6+6 | 10+4 |  |
| FJ9c | clade 1 | Infectious | r/o | Fiji | 6+6/8+4 | 6 | 6 | 8 | 4 | 6+6 | 8+4 |  |
| HMHc1 | clade 1 | Commensal | r/o | USA | 10+4/10+4 | 10 | 4 | 10 | 4 | 10+4 | 10+4 |  |
| HMHc2 | clade 1 | Commensal | r/o | USA | 6+6/6+6 | 6 | 6 | 6 | 6 | 6+6 | 6+6 |  |
| HMHc4 | clade 1 | Commensal | r/o | USA | 6+6/12+4 | 6 | 6 | 12 | 4 | 6+6 | 12+4 |  |
| HMHc5 | clade 1 | Commensal | r/o | USA | 6+9/7+9 | 6 | 9 | 7 | 9 | 6+9 | 7+9 |  |
| HMHc6 | clade 1 | Commensal | r/o | USA | 6+6/9+4 | 6 | 6 | 9 | 4 | 6+6 | 9+4 |  |
| HMHc9 | clade 1 | Commensal | r/o | USA | 6+6/10+4 | 6 | 6 | 10 | 4 | 6+6 | 10+4 |  |
| hp10bt | clade 1 | Commensal | r/o | USA | 6+6/10+4 | 6 | 6 | 10 | 4 | 6+6 | 10+4 |  |
| hp12bt | clade 1 | Commensal | r/o | USA | 6+6/6+6 | 6 | 6 | 6 | 6 | 6+6 | 6+6 |  |
| hp2bt | clade 1 | Commensal | r/o | USA | 6+6/10+4 | 6 | 6 | 10 | 4 | 6+6 | 10+4 |  |
| hp42bt | clade 1 | Commensal | r/o | USA | 6+6/10+4 | 6 | 6 | 10 | 4 | 6+6 | 10+4 |  |
| hp55bt | clade 1 | Commensal | r/o | USA | 6+6/6+6 | 6 | 6 | 6 | 6 | 6+6 | 6+6 |  |
| OD8807 | clade 1 | Infectious | r/o | Great Britain | 6+6/9+4 | 6 | 6 | 9 | 4 | 6+6 | 9+4 |  |
| OD8826 | clade 1 | Infectious | r/o | Great Britain | 6+6/9+4 | 6 | 6 | 9 | 4 | 6+6 | 9+4 |  |
| OD8911 | clade 1 | Infectious | r/o | Great Britain | 10+4/10+4 | 10 | 4 | 10 | 4 | 10+4 | 10+4 |  |
| OD8916 | clade 1 | Infectious | r/o | Great Britain | 6+6/6+6 | 6 | 6 | 6 | 6 | 6+6 | 6+6 |  |
| OD9014 | clade 1 | Infectious | r/o | Great Britain | 6+6/10+4 | 6 | 6 | 10 | 4 | 6+6 | 10+4 | KY569352 |
| rolo-c | clade 1 | Infectious | r/o | USA | 6+6/9+4 | 6 | 6 | 9 | 4 | 6+6 | 9+4 |  |
| sim-c | clade 1 | Infectious | r/o | USA | 6+6/10+4 | 6 | 6 | 10 | 4 | 6+6 | 10+4 |  |
| W104 | clade 1 | Commensal | r/o | New Zealand | 6+6/10+4 | 6 | 6 | 10 | 4 | 6+6 | 10+4 |  |
| W105 | clade 1 | Commensal | r/o | New Zealand | 6+6/10+4 | 6 | 6 | 10 | 4 | 6+6 | 10+4 |  |
| W106 | clade 1 | Commensal | r/o | New Zealand | 6+6/10+4 | 6 | 6 | 10 | 4 | 6+6 | 10+4 |  |
| W107 | clade 1 | Commensal | r/o | New Zealand | 6+6/11+4 | 6 | 6 | 11 | 4 | 6+6 | 11+4 |  |
| W108 | clade 1 | Commensal | r/o | New Zealand | 6+6/10+4 | 6 | 6 | 10 | 4 | 6+6 | 10+4 |  |
| W109 | clade 1 | Commensal | r/o | New Zealand | 6+6/10+4 | 6 | 6 | 10 | 4 | 6+6 | 10+4 |  |
| W111 | clade 1 | Commensal | r/o | New Zealand | 6+6/10+4 | 6 | 6 | 10 | 4 | 6+6 | 10+4 |  |
| W132 | clade 1 | Infectious | r/o | New Zealand | 9+4/9+4 | 9 | 4 | 9 | 4 | 9+4 | 9+4 |  |
| W3 | clade 1 | Infectious | r/o | New Zealand | 6+6/10+4 | 6 | 6 | 10 | 4 | 6+6 | 10+4 |  |
| W43 | clade 1 | Infectious | r/o | New Zealand | 6+6/6+6 | 6 | 6 | 6 | 6 | 6+6 | 6+6 |  |
| W68 | clade 1 | Infectious | r/o | New Zealand | 6+6/10+4 | 6 | 6 | 10 | 4 | 6+6 | 10+4 |  |
| Au39 | clade 1 | Infectious | s/w | New Zealand | 6+8/6+8 | 6 | 8 | 6 | 8 | 6+8 | 6+8 |  |
| Au90 | clade 1 | Infectious | s/w | New Zealand | 6+6/10+4 | 6 | 6 | 10 | 4 | 6+6 | 10+4 |  |
| CH14 | clade 1 | Infectious | s/w | New Zealand | 6+6/9+4 | 6 | 6 | 9 | 4 | 6+6 | 9+4 |  |
| CH42 | clade 1 | Infectious | s/w | New Zealand | 9+4/9+4 | 9 | 4 | 9 | 4 | 9+4 | 9+4 | KY569349 |
| CHB5 | clade 1 | Infectious | s/w | New Zealand | 6+6/9+4 | 6 | 6 | 9 | 4 | 6+6 | 9+4 |  |
| CLB53 | clade 1 | Infectious | s/w | Colombia | 6+6/6+6 | 6 | 6 | 6 | 6 | 6+6 | 6+6 |  |
| FJ26 | clade 1 | Infectious | s/w | Fiji | 6+6/10+4 | 6 | 6 | 10 | 4 | 6+6 | 10+4 |  |
| Au27 | clade 1 | Infectious | sputum | New Zealand | 6+5/6+6 | 6 | 6 | 6 | 5 | 6+6 | 6+5 |  |
| W134 | clade 1 | Infectious | sputum | New Zealand | 6+6/10+4 | 6 | 6 | 10 | 4 | 6+6 | 10+4 |  |
| W59 | clade 1 | Infectious | sputum | New Zealand | 6+4/6+6 | 6 | 4 | 6 | 6 | 6+4 | 6+6 |  |
| HUN122 | clade 1 | Infectious | s | Great Britain | 6+6/9+5 | 6 | 6 | 9 | 5 | 6+6 | 9+5 |  |
| HUN127 | clade 1 | Infectious | s | Great Britain | 6+4/6+4 | 6 | 4 | 6 | 4 | 6+4 | 6+4 |  |
| HUN93 | clade 1 | Infectious | s | Great Britain | 6+6/9+4 | 6 | 6 | 9 | 4 | 6+6 | 9+4 |  |
| HUN95 | clade 1 | Infectious | s | Great Britain | 6+6/9+4 | 6 | 6 | 9 | 4 | 6+6 | 9+4 |  |
| HUN96 | clade 1 | Infectious | s | Great Britain | 6+6/9+4 | 6 | 6 | 9 | 4 | 6+6 | 9+4 |  |
| RIHO10 | clade 1 | Infectious | s | USA | 10+4/10+5 | 10 | 4 | 10 | 5 | 10+4 | 10+5 |  |
| RIHO13 | clade 1 | Infectious | s | USA | 6+6/10+4 | 6 | 6 | 10 | 4 | 6+6 | 10+4 |  |
| RIHO16 | clade 1 | Infectious | s | USA | 11+4/11+4 | 11 | 4 | 11 | 4 | 11+4 | 11+4 | KY569351 |
| RIHO9 | clade 1 | Infectious | s | USA | 10+4/10+4 | 10 | 4 | 10 | 4 | 10+4 | 10+4 |  |
| cpr2.2fec | clade 1 | Commensal | stool | USA | 6+6/9+4 | 6 | 6 | 9 | 4 | 6+6 | 9+4 |  |
| ko-2c | clade 1 | Infectious | stool | USA | 6+6/9+4 | 6 | 6 | 9 | 4 | 6+6 | 9+4 |  |
| AU19 | clade 1 | Infectious | u | New Zealand | 10+4/10+4 | 10 | 4 | 10 | 4 | 10+4 | 10+4 | KY569348 |
| CH35 | clade 1 | Infectious | u | New Zealand | 6+6/10+4 | 6 | 6 | 10 | 4 | 6+6 | 10+4 |  |
| FJ23 | clade 1 | Infectious | u | Fiji | 6+6/9+4 | 6 | 6 | 9 | 4 | 6+6 | 9+4 |  |
| YsM073 | clade 1 | Infectious | u | Malaysia | 6+6/9+4 | 6 | 6 | 9 | 4 | 6+6 | 9+4 |  |
| YsU568 | clade 1 | Infectious | u | Malaysia | 6+6/10+4 | 6 | 6 | 10 | 4 | 6+6 | 10+4 |  |
| YsU649 | clade 1 | Infectious | u | Malaysia | 6+6/9+4 | 6 | 6 | 9 | 4 | 6+6 | 9+4 |  |
| YsU751 | clade 1 | Infectious | u | Malaysia | 6+6/9+4 | 6 | 6 | 9 | 4 | 6+6 | 9+4 |  |
| cfr2.8vag | clade 1 | Commensal | v | USA | 6+6/10+4 | 6 | 6 | 10 | 4 | 6+6 | 10+4 |  |
| cfr2.9vag | clade 1 | Commensal | v | USA | 6+6/10+4 | 6 | 6 | 10 | 4 | 6+6 | 10+4 |  |
| hp11vw | clade 1 | Commensal | v | USA | 6+6/10+4 | 6 | 6 | 10 | 4 | 6+6 | 10+4 |  |
| hp13vw | clade 1 | Commensal | v | USA | 9+4/9+4 | 9 | 4 | 9 | 4 | 9+4 | 9+4 |  |
| hp33vu | clade 1 | Commensal | v | USA | 6+6/10+4 | 6 | 6 | 10 | 4 | 6+6 | 10+4 |  |
| hp33vw | clade 1 | Commensal | v | USA | 6+6/10+4 | 6 | 6 | 10 | 4 | 6+6 | 10+4 |  |
| hp42vp | clade 1 | Commensal | v | USA | 6+6/10+4 | 6 | 6 | 10 | 4 | 6+6 | 10+4 |  |
| CLB42 | clade 1 | Infectious | v | Colombia | 6+6/10+4 | 6 | 6 | 10 | 4 | 6+6 | 10+4 |  |
| var1.10vag | clade 1 | Infectious | v | USA | 7+6/7+6 | 7 | 6 | 7 | 6 | 7+6 | 7+6 |  |
| var1.1vag | clade 1 | Infectious | v | USA | 6+6/10+4 | 6 | 6 | 10 | 4 | 6+6 | 10+4 |  |
| var1.3vag | clade 1 | Infectious | v | USA | 6+6/10+4 | 6 | 6 | 10 | 4 | 6+6 | 10+4 |  |
| var1.4vag | clade 1 | Infectious | v | USA | 6+6/9+4 | 6 | 6 | 9 | 4 | 6+6 | 9+4 | KY569353 |
| var1.8vag | clade 1 | Infectious | v | USA | 6+4/6+4 | 6 | 4 | 6 | 4 | 6+4 | 6+4 |  |
| cfr2.10vul | clade 1 | Commensal | v | USA | 6+6/10+4 | 6 | 6 | 10 | 4 | 6+6 | 10+4 |  |
| cfr2.1vul | clade 1 | Commensal | v | USA | 6+6/10+4 | 6 | 6 | 10 | 4 | 6+6 | 10+4 |  |
| cfr2.3vul | clade 1 | Commensal | v | USA | 6+6/12+4 | 6 | 6 | 12 | 4 | 6+6 | 12+4 |  |
| cfr2.4vul | clade 1 | Commensal | v | USA | 6+6/6+6 | 6 | 6 | 6 | 6 | 6+6 | 6+6 |  |
| hp13vu | clade 1 | Commensal | v | USA | 9+4/9+4 | 9 | 4 | 9 | 4 | 9+4 | 9+4 |  |
| hp31ch | clade 1 | Commensal | r/o | USA | 10+4/10+4 | 10 | 4 | 10 | 4 | 10+4 | 10+4 |  |
| hp31vu | clade 1 | Commensal | r/o | USA | 10+4/10+4 | 10 | 4 | 10 | 4 | 10+4 | 10+4 |  |
| hp31vw | clade 1 | Commensal | r/o | USA | 10+4/10+4 | 10 | 4 | 10 | 4 | 10+4 | 10+4 |  |
| OTG10 | non-clade 1 | Infectious | a | New Zealand | 8+5/9+5 | 8 | 5 | 9 | 5 | 8+5 | 9+5 |  |
| W55 | non-clade 1 | Infectious | a | New Zealand | 8+5/8+5 | 8 | 5 | 8 | 5 | 8+5 | 8+5 |  |
| AU36 | non-clade 1 | Infectious | c | New Zealand | 6+8/9+5 | 6 | 8 | 9 | 5 | 6+8 | 9+5 |  |
| FJ27 | non-clade 1 | Infectious | c | Fiji | 7+6/7+9 | 7 | 6 | 7 | 9 | 7+6 | 7+9 |  |
| RIHO2 | non-clade 1 | Infectious | N | USA | 6+4/6+5 | 6 | 4 | 6 | 5 | 6+4 | 6+5 |  |
| RIHO30 | non-clade 1 | Infectious | N | USA | 6+7/6+31 | 6 | 7 | 6 | 31 | 6+7 | 6+31 | KY569356 |
| RIHO5 | non-clade 1 | Infectious | N | USA | 7+6/7+9 | 7 | 6 | 7 | 9 | 7+6 | 7+9 |  |
| Au134 | non-clade 1 | Infectious | r/o | New Zealand | 6+8/9+5 | 6 | 8 | 9 | 5 | 6+8 | 9+5 |  |
| CLB49 | non-clade 1 | Infectious | r/o | Colombia | 7+4/6+14 | 7 | 4 | 6 | 14 | 7+4 | 6+14 | KY569355 |
| FJ12 | non-clade 1 | Infectious | r/o | Fiji | 8+5/8+5 | 8 | 5 | 8 | 5 | 8+5 | 8+5 |  |
| FJ3 | non-clade 1 | Infectious | r/o | Fiji | 6+6/6+7 | 6 | 6 | 6 | 7 | 6+6 | 6+7 |  |
| Gaymc-c | non-clade 1 | Infectious | r/o | USA | 7+4/6+6 | 7 | 4 | 6 | 6 | 7+4 | 6+6 |  |
| HUN61 | non-clade 1 | Infectious | r/o | Great Britain | 7+9/7+9 | 7 | 9 | 7 | 9 | 7+9 | 7+9 |  |
| HUN68 | non-clade 1 | Infectious | r/o | Great Britain | 6+8/9+5 | 6 | 8 | 9 | 5 | 6+8 | 9+5 |  |
| OD8824 | non-clade 1 | Infectious | r/o | Great Britain | 8+4/8+4 | 8 | 4 | 8 | 4 | 8+4 | 8+4 |  |
| OTG1 | non-clade 1 | Infectious | r/o | New Zealand | 6+9/7+8 | 7 | 8 | 6 | 9 | 7+8 | 6+9 |  |
| OTG18 | non-clade 1 | Infectious | r/o | New Zealand | 6+9/7+9 | 6 | 9 | 7 | 9 | 6+9 | 7+9 |  |
| OTG2 | non-clade 1 | Infectious | r/o | New Zealand | 6+6/7+5 | 6 | 6 | 7 | 5 | 6+6 | 7+5 |  |
| W137b | non-clade 1 | Infectious | r/o | New Zealand | 7+8/7+9 | 7 | 8 | 7 | 9 | 7+8 | 7+9 |  |
| W142 | non-clade 1 | Infectious | r/o | New Zealand | 6+6/8+5 | 6 | 6 | 8 | 5 | 6+6 | 8+5 |  |
| W17 | non-clade 1 | Infectious | r/o | New Zealand | 7+6/7+9 | 7 | 6 | 7 | 9 | 7+6 | 7+9 |  |
| W53 | non-clade 1 | Infectious | r/o | New Zealand | 8+5/8+5 | 8 | 5 | 8 | 5 | 8+5 | 8+5 |  |
| YASM42 | non-clade 1 | Infectious | r/o | Malaysia | 7+4/6+15 | 7 | 4 | 6 | 15 | 7+4 | 6+15 | KY569357 |
| YasU709 | non-clade 1 | Infectious | r/o | Malaysia | 6+8/9+5 | 6 | 8 | 9 | 5 | 6+8 | 9+5 |  |
| Au11 | non-clade 1 | Infectious | s/w | New Zealand | 9+4/7+9 | 9 | 4 | 7 | 9 | 9+4 | 7+9 | KY569347 |
| Au33 | non-clade 1 | Infectious | s/w | New Zealand | 6+6/8+4 | 6 | 6 | 8 | 4 | 6+6 | 8+4 |  |
| CLB44 | non-clade 1 | Infectious | s/w | Colombia | 7+6/7+9 | 7 | 6 | 7 | 9 | 7+6 | 7+9 |  |
| CLB45 | non-clade 1 | Infectious | s/w | Colombia | 8+5/7+9 | 8 | 5 | 7 | 9 | 8+5 | 7+9 |  |
| HUN64 | non-clade 1 | Infectious | s/w | Great Britain | 8+5/8+5 | 8 | 5 | 8 | 5 | 8+5 | 8+5 |  |
| HUN66 | non-clade 1 | Infectious | s/w | Great Britain | 6+6/9+4 | 6 | 6 | 9 | 4 | 6+6 | 9+4 |  |
| OTG6 | non-clade 1 | Infectious | s/w | New Zealand | 9+5/9+5 | 9 | 5 | 9 | 5 | 9+5 | 9+5 | KY569350 |
| HUN123 | non-clade 1 | Infectious | s | Great Britain | 7+9/7+9 | 7 | 9 | 7 | 9 | 7+9 | 7+9 |  |
| HUN91 | non-clade 1 | Infectious | s | Great Britain | 6+24/6+25 | 6 | 24 | 6 | 25 | 6+24 | 6+25 |  |
| HUN92 | non-clade 1 | Infectious | s | Great Britain | 6+6/6+6 | 6 | 6 | 6 | 6 | 6+6 | 6+6 |  |
| YasM1 | non-clade 1 | Infectious | s | Malaysia | 6+7/6+7 | 6 | 7 | 6 | 7 | 6+7 | 6+7 |  |
| CH3 | non-clade 1 | Infectious | u | New Zealand | 6+8/9+5 | 6 | 8 | 9 | 5 | 6+8 | 9+5 |  |
| CH41.1 | non-clade 1 | Infectious | u | New Zealand | 8+4/8+4 | 8 | 4 | 8 | 4 | 8+4 | 8+4 |  |
| YsU123 | non-clade 1 | Infectious | u | Malaysia | 8+4/6+8 | 8 | 4 | 6 | 8 | 8+4 | 6+8 |  |
| YsU363 | non-clade 1 | Infectious | u | Malaysia | 7+4/6+14 | 7 | 4 | 6 | 14 | 7+4 | 6+14 |  |
| YsU63 | non-clade 1 | Infectious | u | Malaysia | 7+4/6+16 | 7 | 4 | 6 | 16 | 7+4 | 6+16 |  |
| CH20 | non-clade 1 | Infectious | v | New Zealand | 6+6/8+4 | 6 | 6 | 8 | 4 | 6+6 | 8+4 |  |
| CH9 | non-clade 1 | Infectious | v | New Zealand | 9+4/9+4 | 9 | 4 | 9 | 4 | 9+4 | 9+4 |  |
| OTG4 | non-clade 1 | Infectious | v | New Zealand | 9+4/7+9 | 9 | 4 | 7 | 9 | 9+4 | 7+9 |  |
| var1.5vag | non-clade 1 | Infectious | v | USA | 6+8/7+9 | 6 | 8 | 7 | 9 | 6+8 | 7+9 | KY569354 |
| vare1.7vul | non-clade 1 | Infectious | v | USA | 6+5/6+6 | 6 | 5 | 6 | 6 | 6+5 | 6+6 |  |
| Au2 | non-clade 1 | Infectious | a/d | New Zealand | 6+7/6+7 | 6 | 7 | 6 | 7 | 6+7 | 6+7 |  |
|  |  |  |  |  |  |  |  |  |  |  |  |  |
| SC5314^e^ | clade 1 | Infectious | Lab | USA | 6+6/6+6 | 6 | 6 | 6 | 6 | 6+6 | 6+6 |  |

^a^ See Schmid et al. (Microbiology 145: 2405-2414; 1999) for more detail on isolates; these authors had assigned strains, by CA3 DNA fingerprinting, to the “general-purpose genotype group”, corresponding to clade 1 (Tavanti et al. 2005, ,J. Clin. Microbiol. 43: 5601-5613; 2005), or to the remainder of the species.

^b^Sites of strain isolation were: anal sites (a), catheter (c), respiratory and oral sites (r/o), skin and wounds (s/w), sterile sites(s), urine (u), vaginal/vulvar (v), abdominal drain (a/d), no information available (N), sputum and stool

^c^ Number of repeat units in both alleles based on calculation of genotyping reading of PCR products’ lengths

^d^Identified in BLASTP search in NCBI website using the SC5314 Ssr1 protein as a query

^e^ The SC5314 strain, having been extensively cultured in the laboratory, was non included in allele frequency calculations.
