# Supplementary material for: Highly mutable tandem DNA repeats generate a cell wall protein variant more frequent in disease-causing Candida albicans isolates than in commensal isolates
Source: PLoS One. 2017 Jun 29;12(6):e0180246. doi: 10.1371/journal.pone.0180246 (PMC5491155; doi:10.1371/journal.pone.0180246)
Supplement: S2 Table — (DOCX) [file pone.0180246.s005.docx]

**S 2 Table. *SSR1* repeat region sizes in published sequences from other strains**

| Strains^a^ | Number of repeats region A^a^ | Number of repeats region B^a^ | Accession number |
| --- | --- | --- | --- |
| 12C | 10 | 6 | PRJNA75209 |
| 19F | 10 | 6 | PRJNA75221 |
| Ca6 | 13 | 5 | PRJNA120431 |
| GC75 | 10 | 8 | PRJNA75223 |
| L26 | 7 | 6 | PRJNA75211 |
| P34048 | 7 | 9 | PRJNA75229 |
| P37037 | 10 | 6 | PRJNA75231 |
| P37039 | 10 | 6 | PRJNA75233 |
| P57055 | 7 | 9 | PRJNA75239 |
| P57072 | 7 | 11 | PRJNA75227 |
| P75010 | 6 | 5 | PRJNA75235 |
| P75016 | 9 | 8 | PRJNA75237 |
| P75063 | 6 | 8 | PRJNA75241 |
| P76067 | 7 | 9 | PRJNA75245 |
| P78042 | 7 | 9 | PRJNA75247 |
| P78048 | 12 | 5 | PRJNA75225 |
| P87 | 9 | 8 | PRJNA75215 |
| P94015 | 6 | 6 | PRJNA75213 |
| *C.dubliniensis* CD36^b^ | 6 | 6 | NC_012866 |

^a^ *SSR1* sequences were identified using BLASTP with the SC5314 sequence as a query and region length was calculated from these sequences

^b^ E value in a BLASTP search with the SC5314 sequence as a query of 4 x 10^-60^; > 75% amino acid sequence identity in non-repetitive regions.
